# Supplementary material for: Exploration of the dynamic interplay between lipids and membrane proteins by hydrostatic pressure
Source: Nat Commun. 2022 Apr 1;13:1780. doi: 10.1038/s41467-022-29410-5 (PMC8975810; doi:10.1038/s41467-022-29410-5)
Supplement: Supplementary file 1 — Supplementary Information [file 41467_2022_29410_MOESM1_ESM.pdf]

Supplementary Information

***

Exploration of the dynamic interplay between lipids and membrane proteins by hydrostatic pressure

Alexandre Pozza,[1,6] François Giraud,[2,6] Quentin Cece,[1,3] Marina Casiraghi,[1,4] Elodie Point,[1] Marjorie Damian,[5] Christel Le Bon,[1] Karine Moncoq,[1] Jean-Louis Banères,[5] Ewen Lescop,[2,*] Laurent J. Catoire[1,*]

[1]Laboratoire de Biologie Physico-Chimique des Protéines Membranaires, UMR 7099, CNRS/Université de Paris, Institut de Biologie Physico-Chimique (IBPC, FR 550), 13 rue Pierre et Marie Curie, 75005 Paris, France; [2]Institut de Chimie des Substances Naturelles (ICSN), CNRS UPR 2301, Université Paris-Saclay, 1 av. de la Terrasse, 91198 Gif-sur-Yvette, France; [3]Present address: Laboratoire Cibles Thérapeutiques et Conception de Médicaments (CiTCoM, UMR 8038, CNRS/Université de Paris), Faculté de Pharmacie, 4 avenue de l'Observatoire, 75270 Paris Cedex 06, France; [4]Present address: Department of Molecular and Cellular Physiology, Stanford University School of Medicine, 279 Campus Drive, 94305 Stanford California, USA; [5]Institut des Biomolécules Max Mousseron (IBMM), Université de Montpellier, CNRS, ENSCM, Pôle Chimie Balard Recherche, 1919 route de Mende, 34293 Montpellier cedex 5, France; [6]These authors equally contributed to this work.

Correspondence: ewen.lescop@cnrs.fr ; laurent.catoire@ibpc.fr

TABLE OF CONTENTS

1. Analysis of translational diffusion of nanodiscs by DOSY (p.2)

2. Supplementary Figures (p.6)

3. Additional References (p.23)

1. Analysis of translational diffusion of nanodiscs by DOSY

The present analysis is related to the observation of the increase of the absolute signal intensity of BLT2 inactive sub-states with pressure in both POPC/POPG and DPPC nanodiscs (*cf.* Fig. 9 in the main text).

Diffusion ordered NMR spectroscopy (DOSY) allows measuring translational diffusion coefficient of compounds, which reports on their molecular size. In DOSY experiments, a pair of encoding/decoding gradients sandwiches a diffusion delay Δ and NMR signal intensity is recorded for increasing gradient strengths. In the experiments presented herein, a Bipolar Pulse Pair Stimulated Echo NMR pulse sequence was modified to allow water suppression by excitation sculpting and by addition of a 5 ms delay to attenuate longitudinal eddy current effects as implemented previously [1]. The diffusion delay Δ was set to 300 ms. The coding/decoding gradients had a length δ of 1500 ms, and gradient intensity g was varied from 5 to 90% of the maximal value (50 G·cm^{-1}). DOSY spectra were processed using Topspin and Dynamics Center software (Bruker BioSpin) and analyzed with one or two-component Stejskal-Tanner equation [2]:

$$f(g) = I_{01}e^{-\gamma^2 g^2 \delta^2 (\Delta - \delta/3) \times D_1} + I_{02}e^{-\gamma^2 g^2 \delta^2 (\Delta - \delta/3) \times D_2}$$

where I_{0i} ($i = 1{,}2$) is the intensity at 0% gradient strength, γ the ^{1}H gyromagnetic ratio, g the gradient strength, δ the gradient length, Δ the diffusion delay and D_i the translational diffusion coefficients (m^2·s^{-1}).

The DOSY curves extracted for CH_2 and CH_3 phospholipid signals are shown in Figure A at 1 and 2500 bar and could be fitted to single or double-component Stejskal-Tanner equations to obtain the translational diffusion coefficients. Table A sums up the D coefficient measured for phospholipid CH_2 and CH_3 signals for different nanodisc compositions (phospholipid/cholesterol mixtures, and presence or not of BLT2 and the agonist LTB4). At 1 bar, cholesterol signals were weak and superimposed with CH_2 and CH_3 phospholipid signals and accurate diffusion coefficients could not be obtained. At 2500 bar, cholesterol signals were clearly visible in ^{1}H dimension and analysis of their diffusion-encoded signals revealed similar hydrodynamic properties for cholesterol and phospholipid signals (data not shown), which confirmed that the NMR-visible cholesterol is embedded in nanodiscs. Of note, DOSY experiments are dominated in the 0–2 ppm region by the strong signals of phospholipids and cholesterol at natural ^{1}H abundance and this region does not contain detectable contributions from the highly deuterated BLT2 protein, for which we could not measure diffusion properties.

Table A shows that the diffusion coefficients of phospholipid CH_2 and CH_3 were constant in all tested samples at a value around $5 \cdot 10^{-11}$ m^2·s^{-1}. This value is intermediate between diffusion coefficients measured for small molecules ($\sim 10^{-10}$ m^2·s^{-1}) and 100 nm diameter liposomes ($\sim 10^{-12}$ m^2·s^{-1}) and is therefore in agreement with the formation of nanodiscs under all conditions. For several samples, a single component Stejskal-Tanner equation was sufficient to fit experimental curves suggesting that all phospholipids were collectively and homogenously part of nanodiscs and no free phospholipid was present in these NMR samples. For other samples, a second component ($\sim 10^{-10}$ m^2·s^{-1}) was required to properly fit the data, suggesting that a fraction of phospholipids or LTB4 was free in solution. Due to the very different transverse relaxation properties of rapidly and

slowly diffusing species, it is not possible to directly convert the I% value from Table A into the relative molar populations of rapidly and slowly diffusing species.

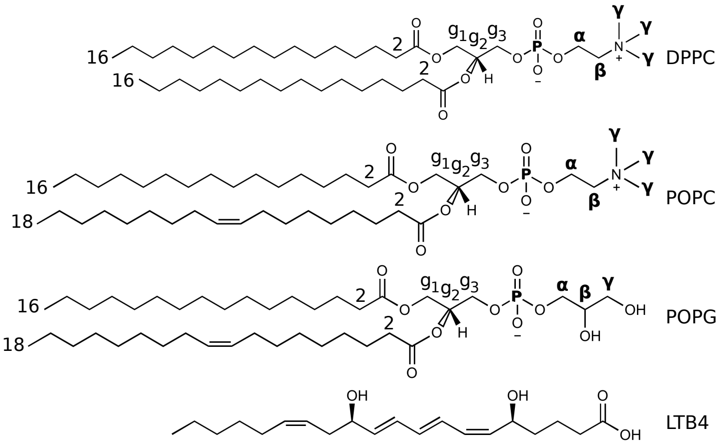

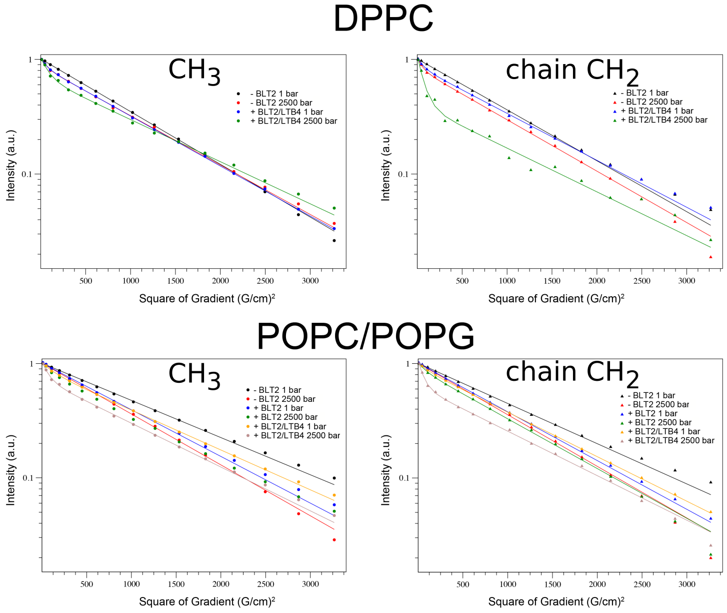

Fig. A. DOSY experiments. The curves come from the integrals in the ^{1}H regions of chain CH_2 (∼1.2 ppm) and CH_3 (∼0.8 ppm) groups from DPPC and POPC/POPC phospholipids in the respective nanodiscs. DOSY data was collected at 25°C and 950 MHz ^{1}H frequency at 1 and 2500 bar for nanodiscs without BLT2 (−BLT2) and in the presence of the receptor (+BLT2) and also in the absence or presence of the LTB4 ligand. The continuous lines show the best-fit curve with single or double component Stejskal-Tanner equations using the parameters summed up in Table A displayed on page 5. Source data are provided as a Source Data file.

The diffusion coefficient values were only slightly perturbed (±10%) by pressure suggesting that the hydrodynamic properties of nanodiscs were essentially preserved at 1 and 2500 bar. The diffusion coefficient D is related to solution viscosity η and particle radius r via the Stokes Einstein law D=kT/6$\pi\eta$r (k is the Boltzmann constant). Pure water viscosity slightly increases (+6%) from 890 μPa·s to 948 μPa·s when pressure increases

from 1 bar to 2500 bar (see Figure B). Therefore, assuming unmodified particle size at 2500 bar, ∼6% slower diffusion would be expected at 2500 bar compared to 1 bar on the basis of viscosity change. Hence, the DOSY-derived values suggest that pressure has minimal impact on nanodisc apparent size.

We further interpreted the large (×3 to 5 in intensity, Fig. 9 in the main text, or ×2 in volume, Fig. S15C,E) signal intensity increase for BLT2 Ile and Met methyl groups in ^{1}H,^{13}C SOFAST–HMQC spectra in light of nanodisc hydrodynamic properties. At the high nanodisc concentrations used here, we could expect potential self-association between nanodiscs, leading to line-broadening and slow translational diffusion. Since pressure is known to reduce intermolecular interaction, we hypothesized that pressure could reduce nanodisc self-association resulting in reduced tumbling correlation time (τ_c), faster translational diffusion, and more favorable NMR methyl relaxation rates at 2500 bar compared to 1 bar. To test this idea, and since rotational τ_c could not be directly measured here due to low signal-to-noise ratio for ^{15}N or methyl ^{1}H relaxation experiments, we exploited the following relationship between the translational (D) and rotational (D_r, or correlation time τ_c=1/(6D_r)) diffusion coefficients: $D\times\tau_c$=2r^2/9, based on the Stokes Einstein Debye law: D_r=kT/(8$\pi\eta$r^3) or τ_c=4$\eta\pi$r^3/(3kT). Assuming that the nanodisc particle has constant shape upon pressure, the DOSY data indicates that the apparent tumbling correlation time of nanodiscs is not highly perturbed (within ±10%) by pressure, and that the dramatic methyl receptor and cholesterol signal increase observed from 1 to 2500 bar is not due to a change in tumbling time. We propose that changes in internal dynamics within nanodisc particles or the receptor might be alternative explanations. This reduced line-broadening could be due to pressure-induced changes in populations and/or timescales of motion in the exchange processes. (see Fig. 9 and also $^{13}CH_3$-δ_1-Ile signals in Fig. S12 & S13 and $^{13}CH_3$-ϵ-Met signals in Fig. S9).

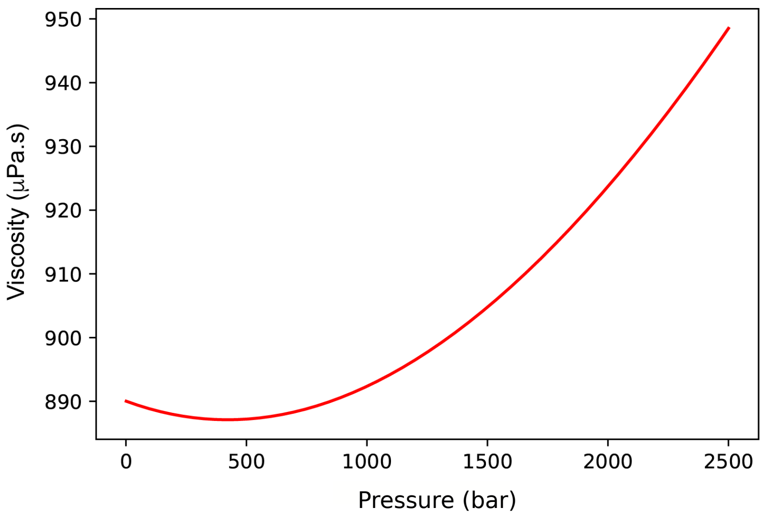

Fig. B. Evolution of viscosity of pure water from 1 to 2500 bar at 25°C. Values obtained from CoolProp [3].

CHAIN CH_2		1 BAR $D_{nanodisc}$ (10^{-10} m^2·s^{-1})	1 BAR $D_{secondary}$ (10^{-10} m^2·s^{-1})	1 BAR I%	2500 BAR $D_{nanodisc}$ (10^{-10} m^2·s^{-1})	2500 BAR $D_{secondary}$ (10^{-10} m^2·s^{-1})	2500 BAR I%
DPPC	−BLT2	0.54 ± 0.01			0.54 ± 0.02	9.5 ± 3.0	20
	+BLT2/LTB4	0.50 ± 0.02	7.7 ± 2.9	16	0.46 ± 0.13	7.0 ± 2.5	63
POPC/POPG	−BLT2	0.43 ± 0.02			0.54 ± 0.01		
	+BLT2	0.52 ± 0.01			0.53 ± 0.03	6.0 ± 3.7	12
	+BLT2/LTB4	0.47 ± 0.01	4.2 ± 0.9	9	0.47 ± 0.03	8.1 ± 1.4	41

CH_3		1 BAR $D_{nanodisc}$ (10^{-10} m^2·s^{-1})	1 BAR $D_{secondary}$ (10^{-10} m^2·s^{-1})	1 BAR I%	2500 BAR $D_{nanodisc}$ (10^{-10} m^2·s^{-1})	2500 BAR $D_{secondary}$ (10^{-10} m^2·s^{-1})	2500 BAR I%
DPPC	−BLT2	0.56 ± 0.01			0.52 ± 0.02	8.5 ± 3.4	15
	+BLT2/LTB4	0.53 ± 0.01	8.5 ± 2.0	15	0.44 ± 0.04	5.7 ± 1.8	33
POPC/POPG	−BLT2	0.40 ± 0.01			0.54 ± 0.01		
	+BLT2	0.50 ± 0.02			0.47 ± 0.03	3.5 ± 1.4	18
	+BLT2/LTB4	0.42 ± 0.02	3.4 ± 1.2	14	0.47 ± 0.03	6.8 ± 1.8	30

Table A. Translational diffusion coefficients $D_{nanodisc}$. $D_{nanodisc}$ has been evaluated for the chain CH_2 and CH_3 groups of phospholipids measured in DPPC or POPC/POPG nanodiscs and in presence or absence of BLT2 and LTB4 at 1 and 2500 bar. When two components were required for a proper fit, the second diffusion coefficient value $D_{secondary}$ and the relative fraction I% of the rapidly diffusing component (calculated as $I_{02}/(I_{01}+I_{02})$) are shown. The rapidly diffusing component likely corresponds to isolated lipid molecules, smaller phospholipid entities, or, when present in the sample, the ligand LTB4.

2. Supplementary Figures

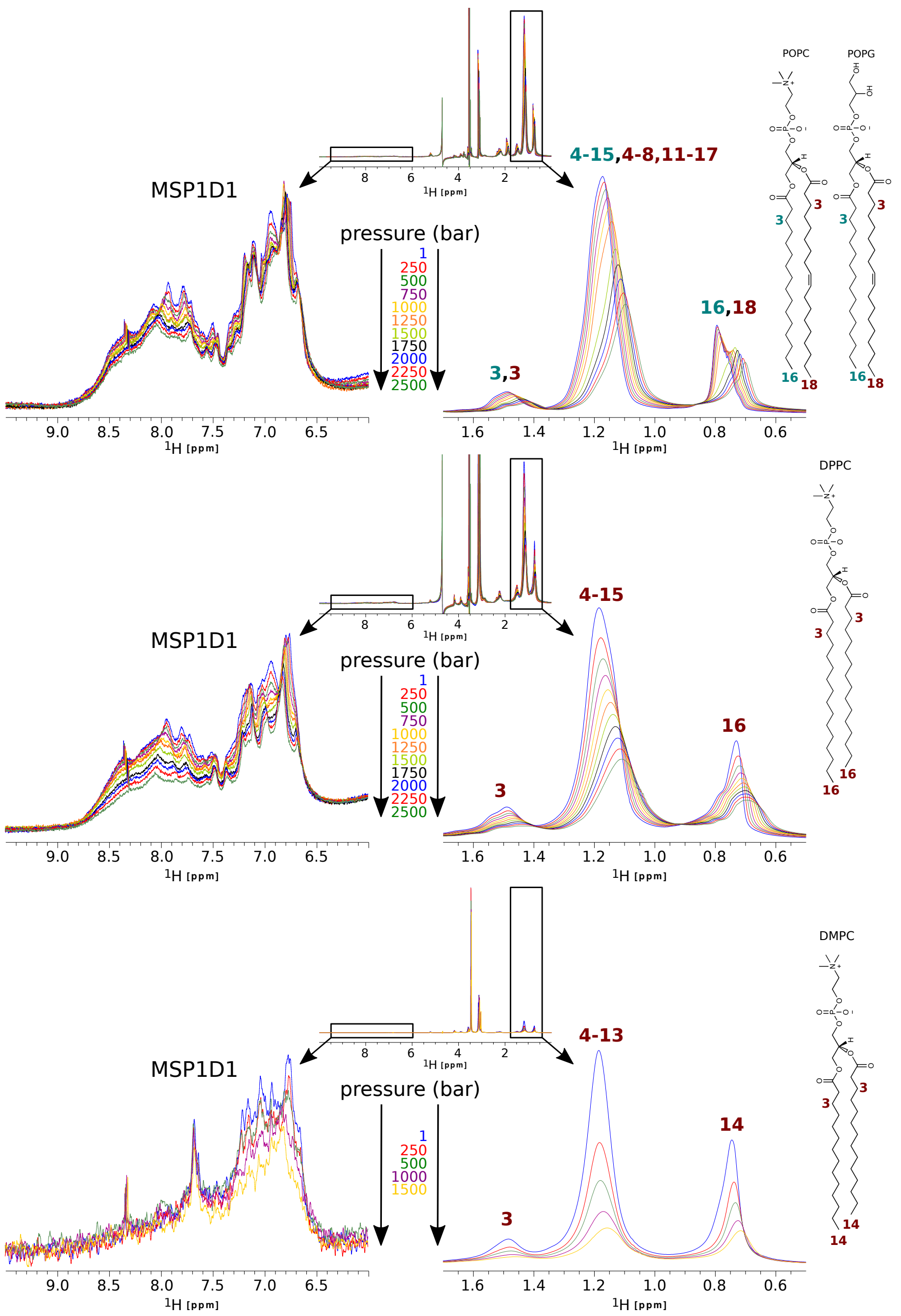

Fig. S1. Illustration of the barotropic evolution of some lipoprotein and lipid NMR signals. The NMR signals of lipoprotein MSP1D1 and lipids are displayed on the *left* and *right*, respectively, in the absence of the receptor along the pressure ramp at 25°C observed in one-dimensional ^{1}H (950 MHz ^{1}H Larmor frequency) NMR spectra. (*Top*), (*Middle*) and (*Bottom*) are POPC/POPG, DPPC and DMPC receptor-free nanodiscs, respectively.

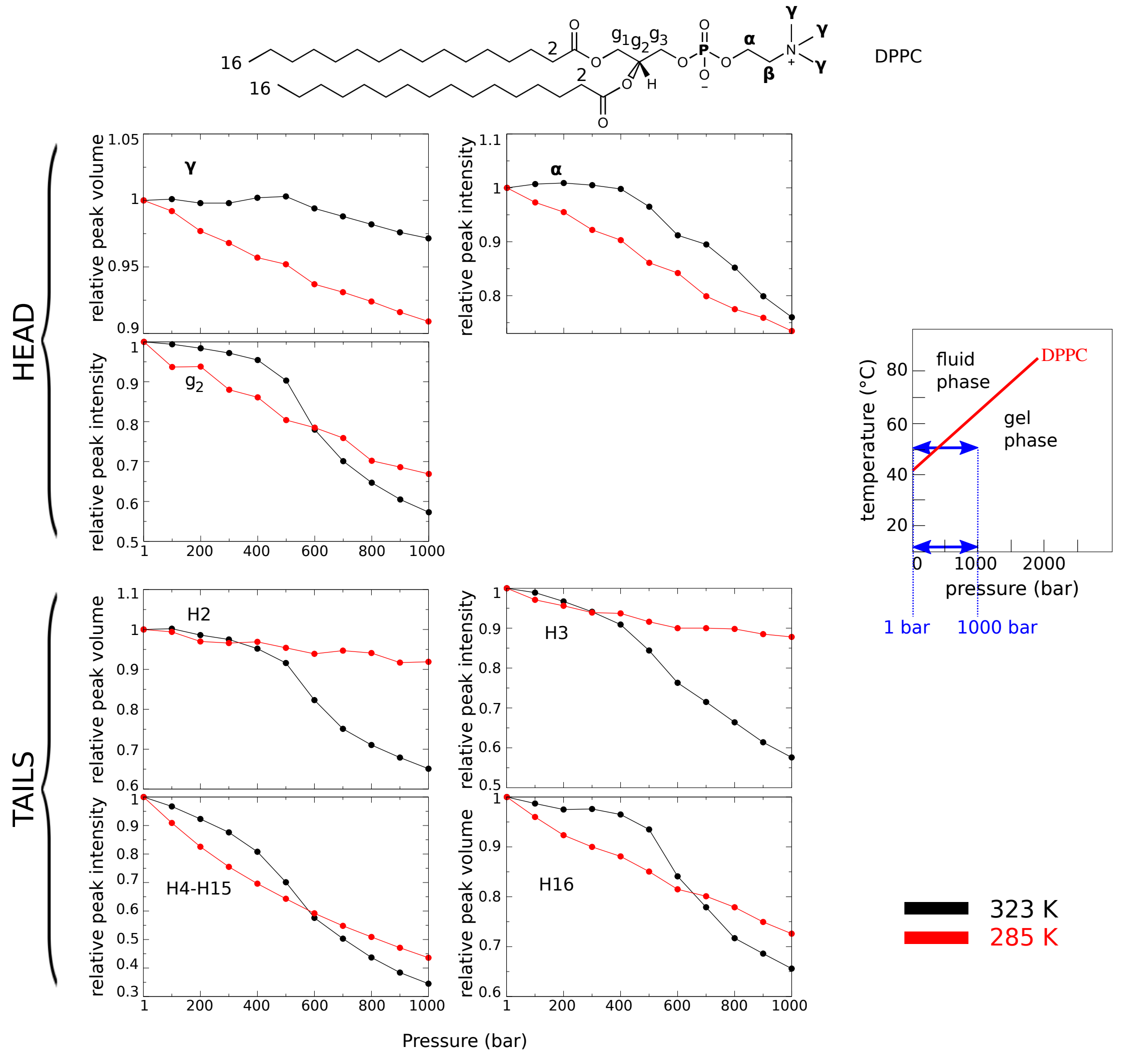

Fig. S2. Barotropic fluid-to-gel phase transition of DPPC molecules confined in MSP1D1 nanodiscs. Diagrams depict two superimposed ramps in pressure performed at 323 (in *black*) and 285K (in *red*) for various protons based on 1D ^{1}H NMR experiments (^{1}H Larmor frequency = 600 MHz). β, g_1 and g_3 protons are not represented because of a too low spectral resolution. As indicated by the two double arrows colored in *blue* on the P/T phase diagram [4] on the right, only experiments performed at 323K can cause a phase transition. Source data are provided as a Source Data file.

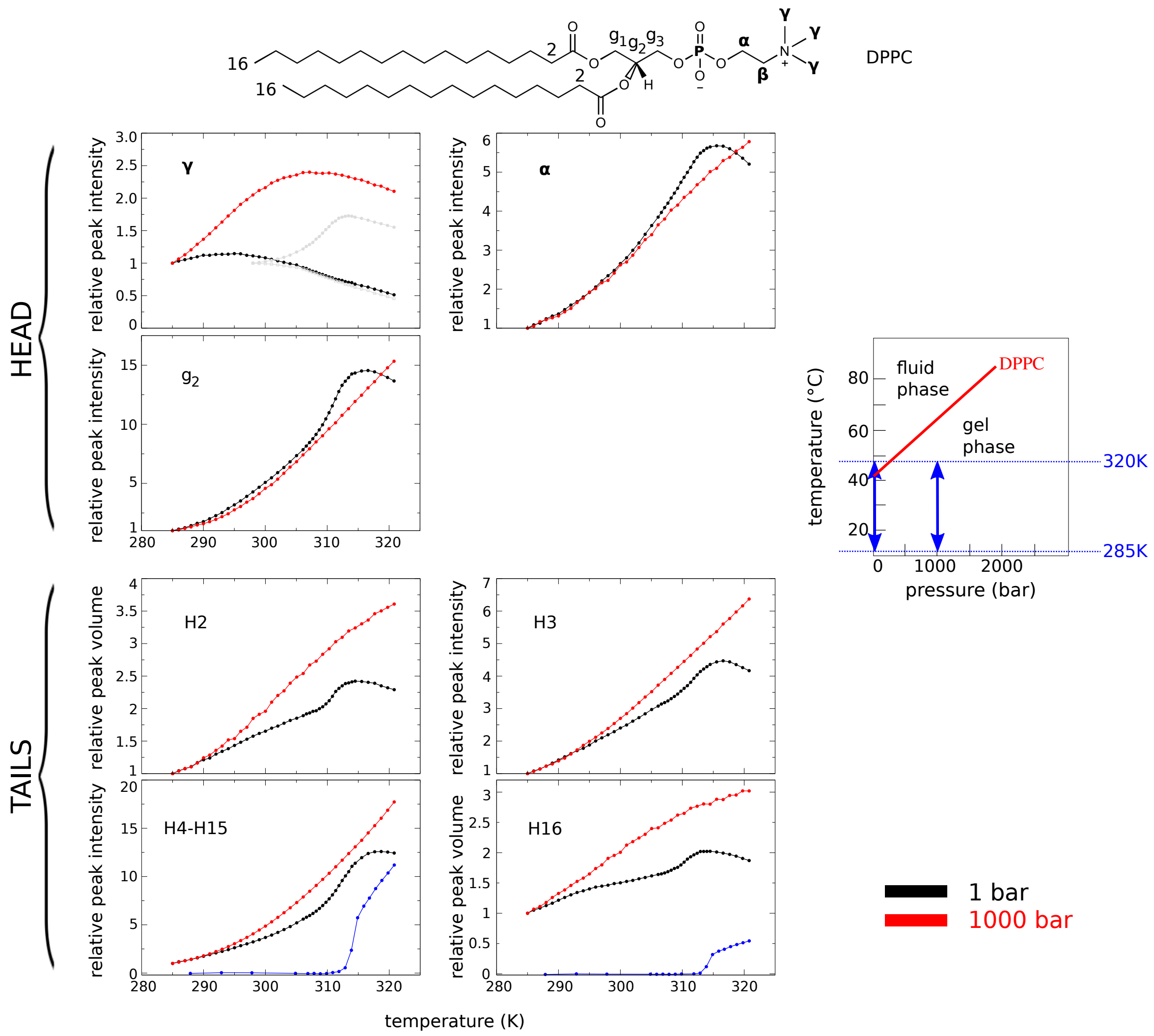

Fig. S3. Thermotropic gel-to-fluid phase transition of DPPC molecules confined in MSP1D1 nanodiscs. Diagrams depict superimposed ramps in temperature between 285 and 320K performed at 1 (in *black*) and 1000 bar (in *red*) for various protons based on 1D ^{1}H NMR experiments (^{1}H Larmor frequency = 600 MHz). β, g_1 and g_3 protons are not represented because of a too low spectral resolution. As indicated by the two double arrows colored in *blue* on the P/T phase diagram [4] on the right, only experiments performed at 1 bar can cause a phase transition. In the γ diagram (upper left), the signal at 1 bar becomes doubled at temperature > 298K (represented by two grey curves in addition to the black curve that represents the sum of the two signals). In the H4-H15 and H16 diagrams, the blue curves that represent the thermotropic gel-to-fluid transitions at 1 bar of these protons in ~100 nm liposomal vesicles observed by solution-state NMR (from ref [5]) have been added for the sake of comparison. Note that NMR peak intensities decreased at temperatures >315 K. This corresponded to a slight irreversible sample precipitation at these high temperatures. This was not visible at 1000 bar, suggesting that DPPC nanodiscs in the fluid phase may tend to aggregate at such temperatures. Source data are provided as a Source Data file.

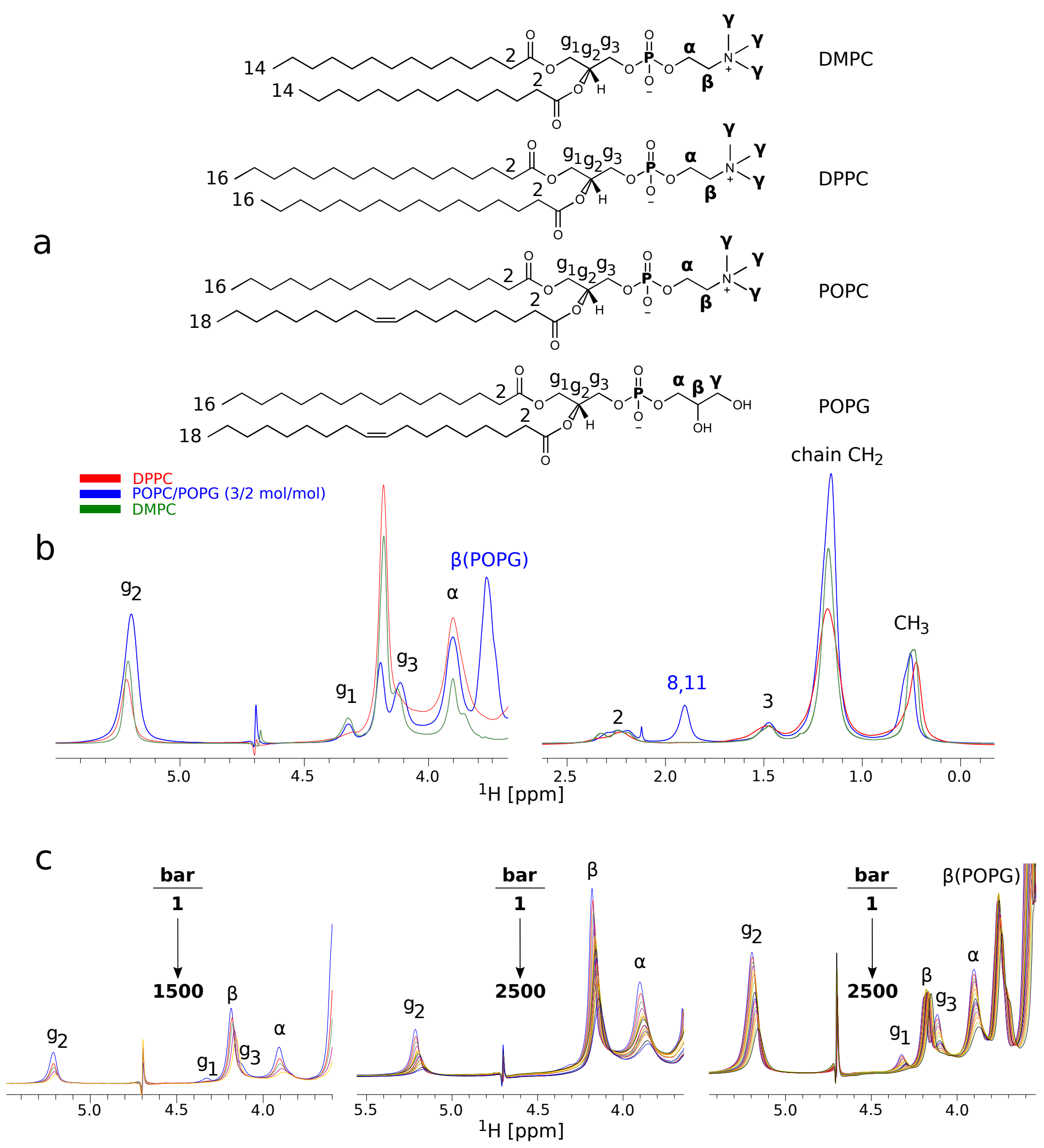

Fig. S4. One-dimensional ^{1}H NMR signals of lipid in nanodiscs. (**a**) Lipid primary structures. (**b**) ^{1}H NMR assignments at 25°C of DMPC (*green*), DPPC (*red*) and POPC/ POPG (in *blue*) heads and tails based on the literature (*e.g.* ref. [6]). The g_1 and g_3 ^{1}H signals of DPPC are not visible due to the signal of α protons which is broader than equivalent protons in POPC and POPG. (**c**) Illustration of the evolution of ^{1}H NMR signals of the lipid heads upon pressurization: (*left*) DMPC, (*center*) DPPC, and (*right*) POPC/POPG (example in the g_2 to α chemical shift region).

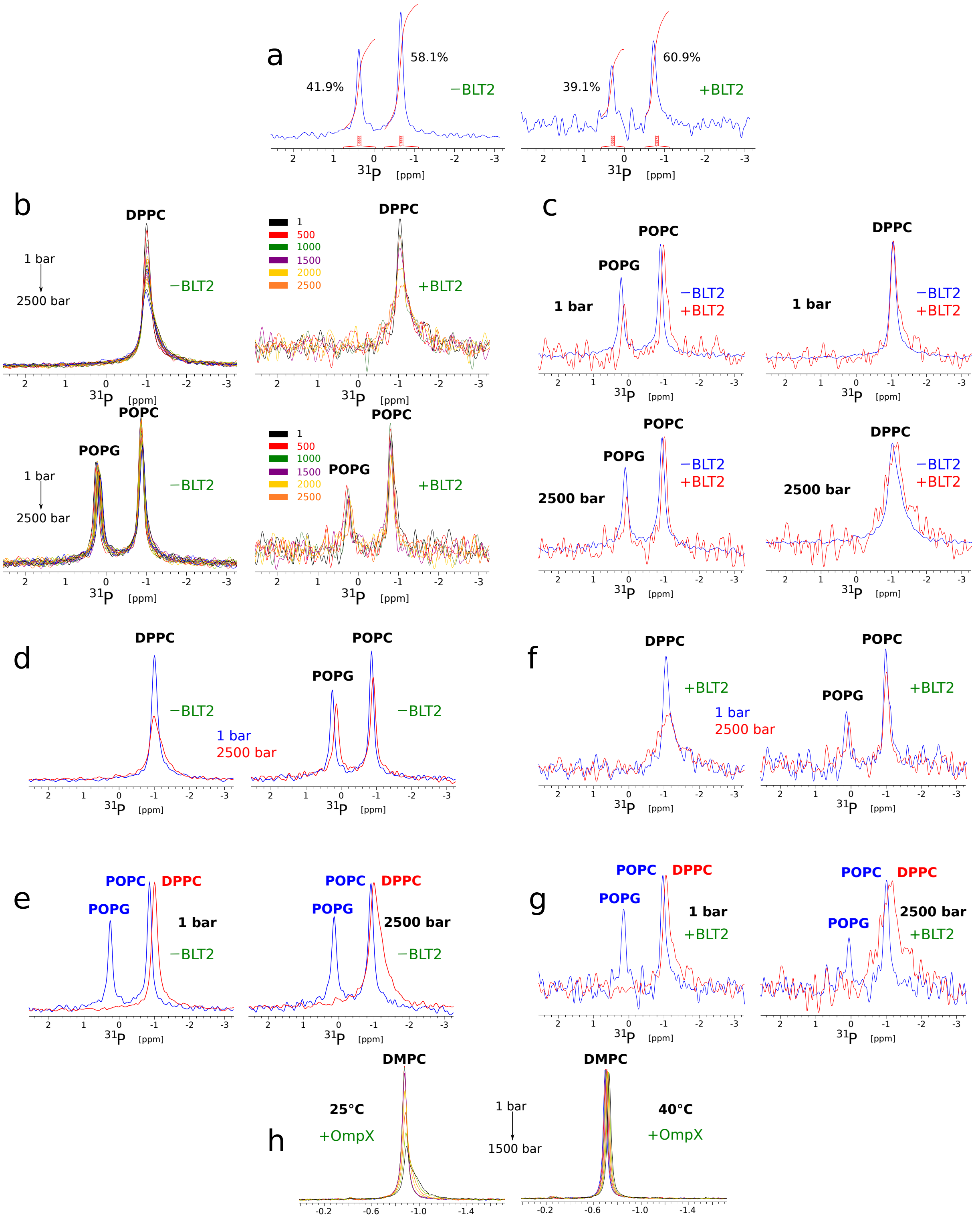

a
41.9%
58.1%
−BLT2
39.1%
60.9%
+BLT2
b
DPPC
1 bar
2500 bar
−BLT2
1
500
1000
1500
2000
2500
+BLT2
POPC
POPG
c
1 bar
−BLT2
+BLT2
2500 bar
d
1 bar
2500 bar
e
f
g
h
DMPC
25°C
+OmpX
1500 bar
40°C
31P [ppm]

Fig. S5. One-dimensional ^{31}P NMR (243 MHz). (**a**) Proportion of POPC *vs.* POPG based on signal integration in the case of receptor-free (*left*) or BLT2-containing (*right*) nanodiscs. The integrals indicate ratio close to the original ratio used at the time of the reconstitution process (3/2 POPC/POPG mol/mol). (**b**) Barotropic evolution of ^{31}P signals of DPPC (*Top*) and POPC/POPG (*Bottom*) receptor-free (*left*) and BLT2-containing (*right*) nanodiscs. (**c**) Comparisons at 1 (*Top*) and 2500 (*Bottom*) bar between receptor-free (in *blue*) and BLT2-containing (in *red*) POPC/POPG (*left*) and DPPC (*right*) nanodiscs. (**d**) Comparisons between 1 (in *blue*) and 2500 (in *red*) bar for DPPC (*left*) and POPC/POPG (*right*) receptor-free nanodiscs. (**e**) Comparisons at 1 (*left*) and 2500 (*right*) bar between DPPC (*red*) and POPC/POPG (*blue*) receptor-free nanodiscs. (**f**) Same as **d** but in the presence of *apo* BLT2. (**g**) Same as **e** but in the presence of *apo* BLT2. (**h**) Barotropic evolutions of ^{31}P signals of DMPC in the presence of OmpX at 25 (*left*) and 40°C (*right*).

$\longleftarrow$

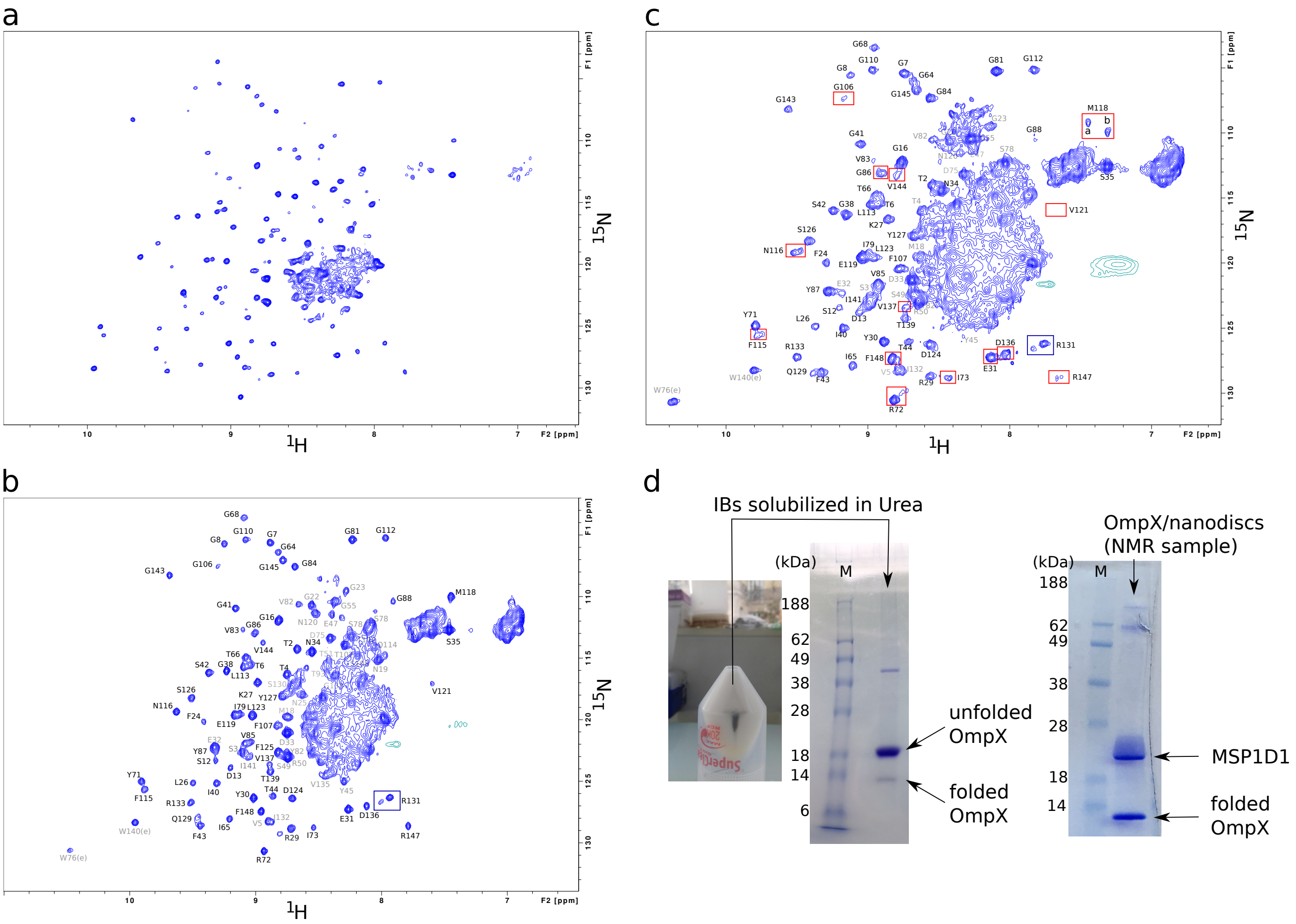

Fig. S6. 2D ^{1}H,^{15}N NMR spectra of OmpX and assesment of the quality of the purification and of the NMR sample by SDS-PAGE electrophoresis. OmpX ^{1}H,^{15}N correlation spectra collected at 40°C (panels **a** and **b**) and 25°C (panel **c**). ^{15}N BEST–TROSY [7] (panel **a**) and ^{15}N SOFAST–HMQC [8] (panels **b** and **c**) experiments were used. At 40°C, the SOFAST-HMQC performed well for well resolved signals but showed poor resolution for cross-peaks in the center of the spectrum. This is likely due to the fast ^{1}H^{N}–solvent exchange for the flexible residues in OmpX loops at the high temperature used (40°C) at pH 7.3. The peak assignment was obtained from the assignment data available for OmpX in nanodisc (BMRB Code 18796, [9]). The assignment transfer from 25°C to 40°C was facilitated by a series of experiments collected at intermediate temperatures (from 25 to 40°C every 5°C). In grey are residues that could not be unambiguously assigned and have been discarded in the analysis. Indole ^{1}H of Trp residues were also excluded. In panel **c**, red rectangles indicate residues that present splitted signals at 25°C but not at 40°C. By contrast, the splitted signal of R131 is present at both temperature and is framed in blue. (**d**) Assesment of the purity and degree of folding of urea-solubilized OmpX inclusion bodies (IBs) and NMR sample by acrylamide gel (12%) electrophoresis.

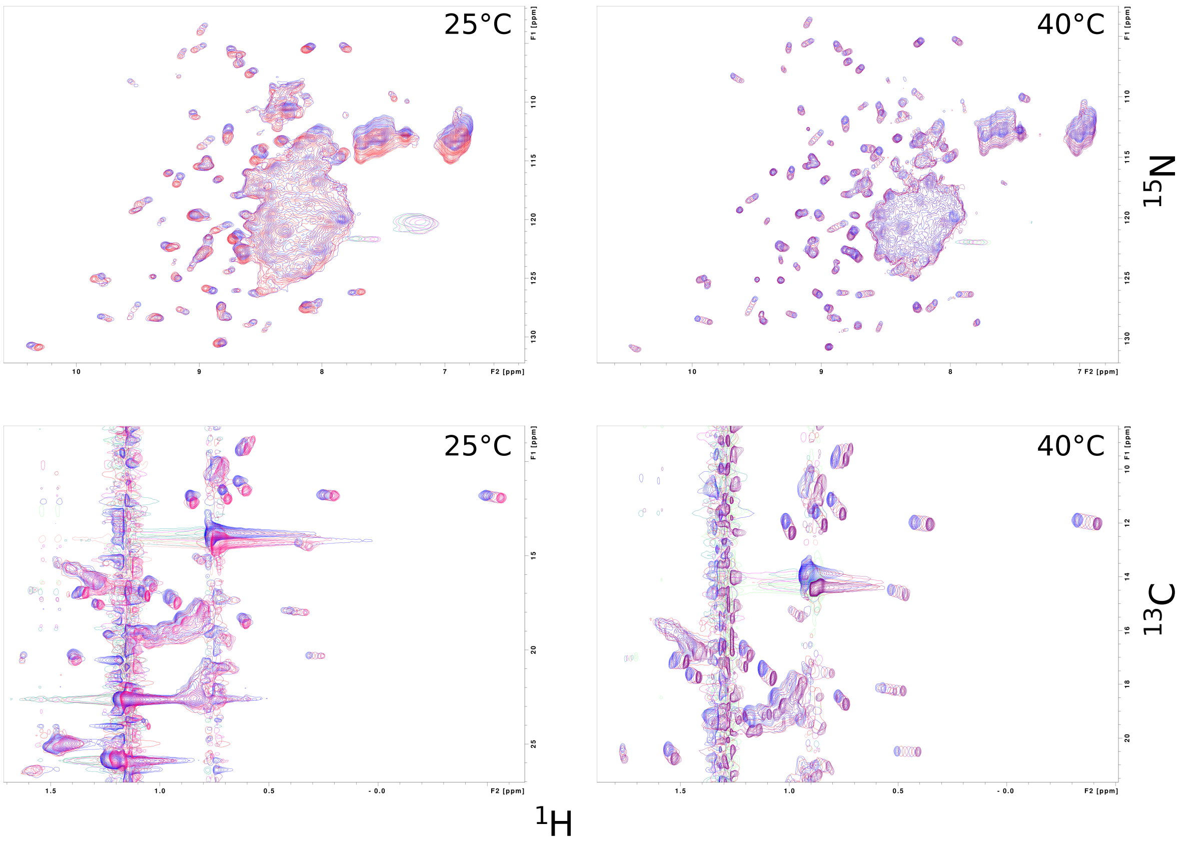

Fig. S7. Barotropic evolution of $^{1}H^{N}$,^{15}N backbone (*Top*) and $^{13}CH_3$ (*Bottom*) NMR signals of OmpX in DMPC nanodiscs at 25 and 40°C.

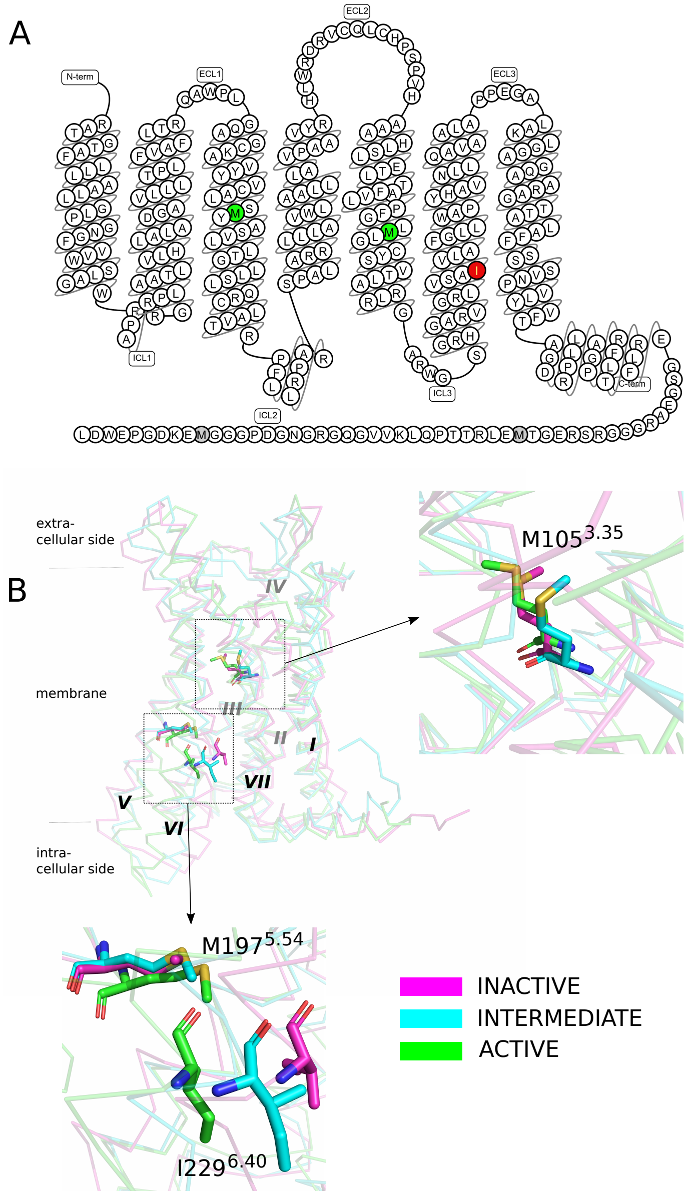

Fig. S8. Localization of the three trans-membrane $^{13}CH_3$ NMR reporters in human BLT2 receptors. (**a**) Snake diagram of the receptor generated with GPCRdb web tools [10]. The unique isoleucine I229$^{6.40}$ and the two transmembrane methionines M105$^{3.35}$ and M197$^{5.54}$ are indicated in *red* and *green*, respectively. The three extramembrane Met residues (M1, not shown, and M325 and M349 colored in *grey* in the Cter region) were mutated to alanine residues to ease the observation of M105 and M197 NMR signals. (**b**) Three superimposed GPCRdb BLT2 receptor homology models (*inactive* in *magenta*, *intermediate* in *cyan* and *active* in *green* conformations). Roman letters indicate transmembrane helices (in *black* those visible on this side of the receptor and in *grey* the helices locaded on the other side).

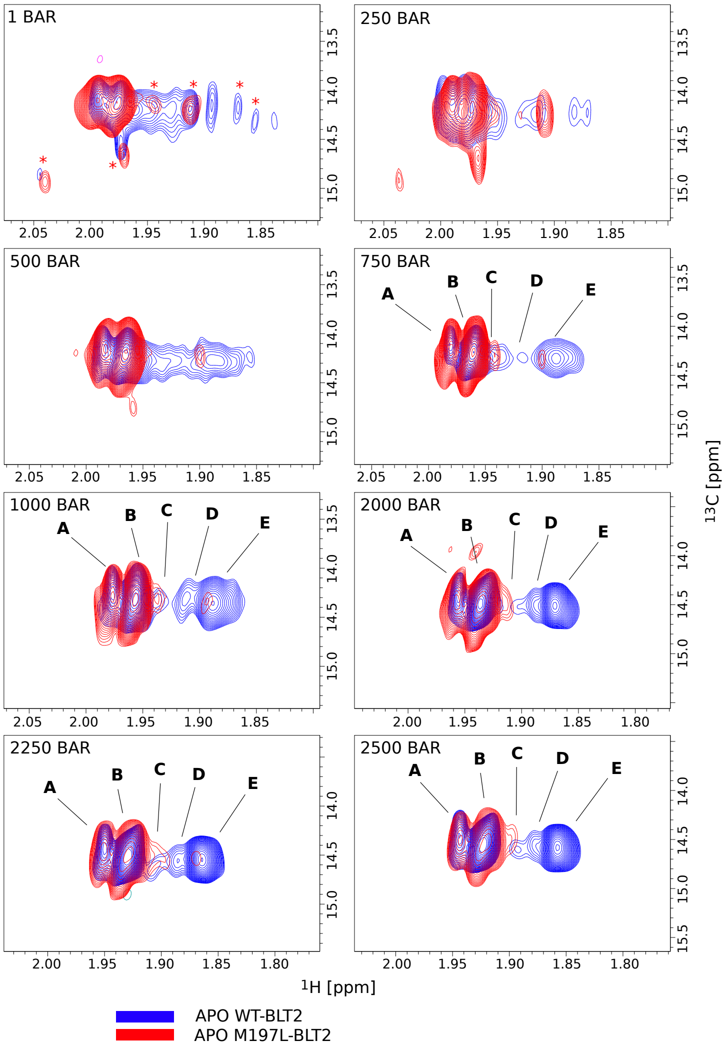

Fig. S9. $^{13}CH_3$ NMR assignment of the two trans-membrane Met residues of BLT2. Comparison of ϵ-$^{13}CH_3$-Methionine NMR signals between the wild-type BLT2 (in *blue*) and M197L-BLT2 (in *red*) receptors in POPC/POPG nanodiscs observed in two superimposed two-dimensional ^{1}H,^{13}C–SOFAST–HMQC (950 MHz ^{1}H Larmor frequency) NMR spectra at 25°C. These experiments allow the assignment of the two transmembrane methionine signals. The barometric behavior of I229$^{6.40}$, M105$^{3.35}$ and M197$^{5.54}$ $^{13}CH_3$ sub-states have been studied from 500 to 2500 and 750 to 2500 bar, respectively (see Fig. 7), because at pressures $\leq$500 bar there are too many sub-states (indicated by a red asterisk at 1 bar) and/or the signal-to-noise ratio is too low which complicates the evaluation of the populations observed all along the pressure ramp at 750 bar and above.

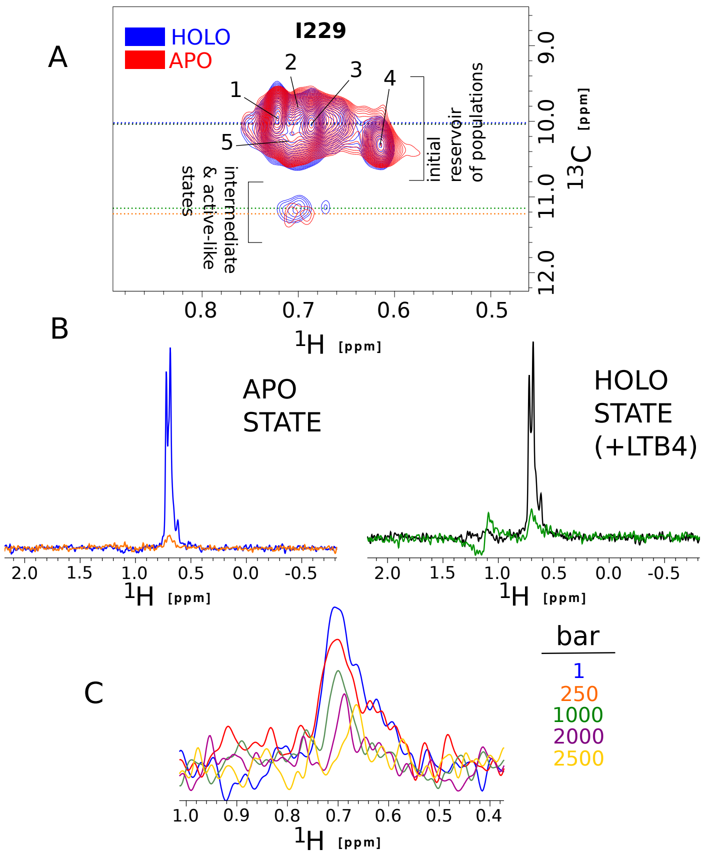

Fig. S10. Comparison of I229 lowly-populated NMR signals between the *apo* and *holo* states of BLT2 (+LTB4 at a no saturating concentration, see Methods section). (**a**) Close-up in the δ_1-$^{13}CH_3$-I229$^{6.40}$ region in two superimposed two-dimensional ^1H,^{13}C–SOFAST–HMQC (950 MHz ^1H Larmor frequency) NMR spectra of BLT2 in POPC/POPG nanodiscs in the *apo* (contour plot in *red*) and *holo* (in *blue*) states. The colored dotted lines indicate ^{13}C frequencies at which rows along the ^1H dimension have been extracted and compared in **b**. In this example, the experiments have been performed at 750 bar. Numbers refer to the high-populated sub-states described in Fig. 7 of the main text. (**b**) Comparison of the intensities of rows extracted at the most intense signals of highly populated states (in *blue* and *black* in the *apo* and *holo* states, respectively) with rows that correspond to the lowly populated states (in *orange* and *green*, in the *apo* and *holo* states, respectively). (**c**) Illustration of the decrease of the lowly-populated states along the pressure ramp in the presence of the agonist LTB4. This has to be compared with the ×3 to 5 increase in signal intensity (Fig. 9 of the main text) or the ×2 increase in signal volume (Fig. S15) for the inactive states at 2500 bar.

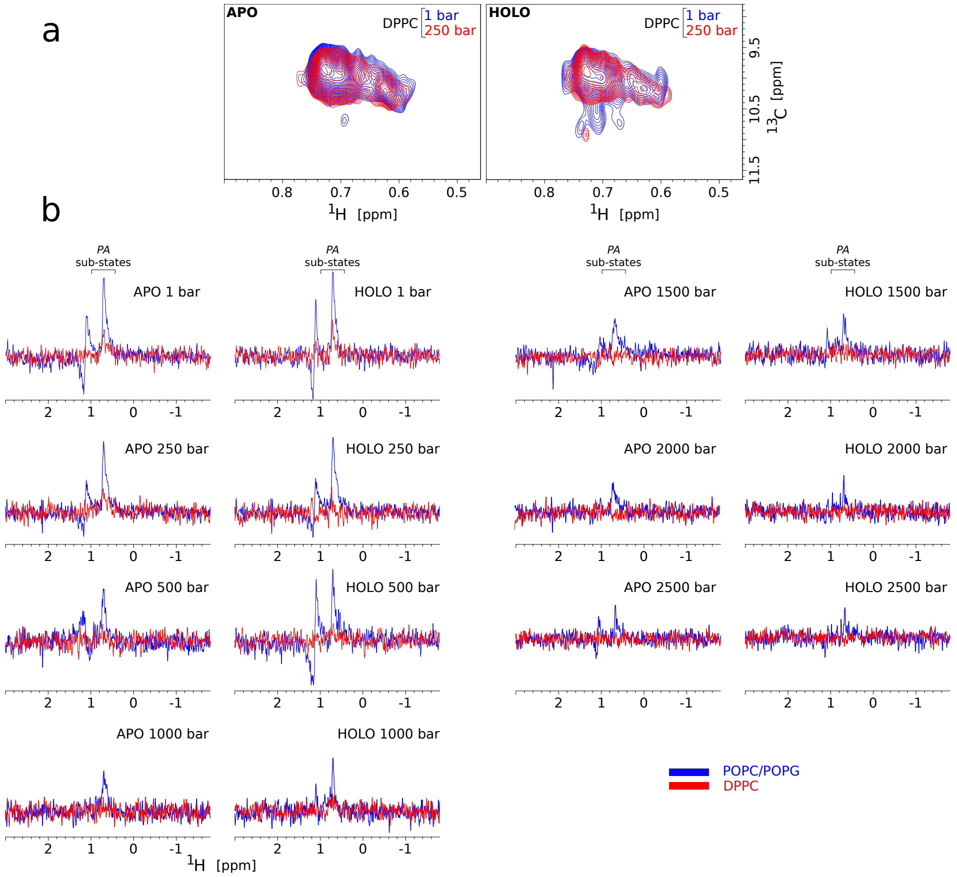

Fig. S11. I229 Pre-Active (*PA*) sub-states in DPPC nanodiscs. (**a**) Close-ups in the $^{13}CH_3$-δ_1-I229$^{6.40}$ region of two superimposed ^{1}H,^{13}C SOFAST–HMQC experiments recorded at atmospheric pressure (in *blue*) and 250 bar (in *red*) with *apo* (*left*) and *holo* (*right*) BLT2 in DPPC nanodiscs. (**b**) Extracted ^{1}H rows at the ^{13}C frequency of *PA* peaks from 2D ^{1}H,^{13}C SOFAST–HMQC experiments recorded with BLT2 in POPC/POPG (in *blue*) or DPPC (in *red*) in the *apo* (*left*) or *holo* (*right*) states.

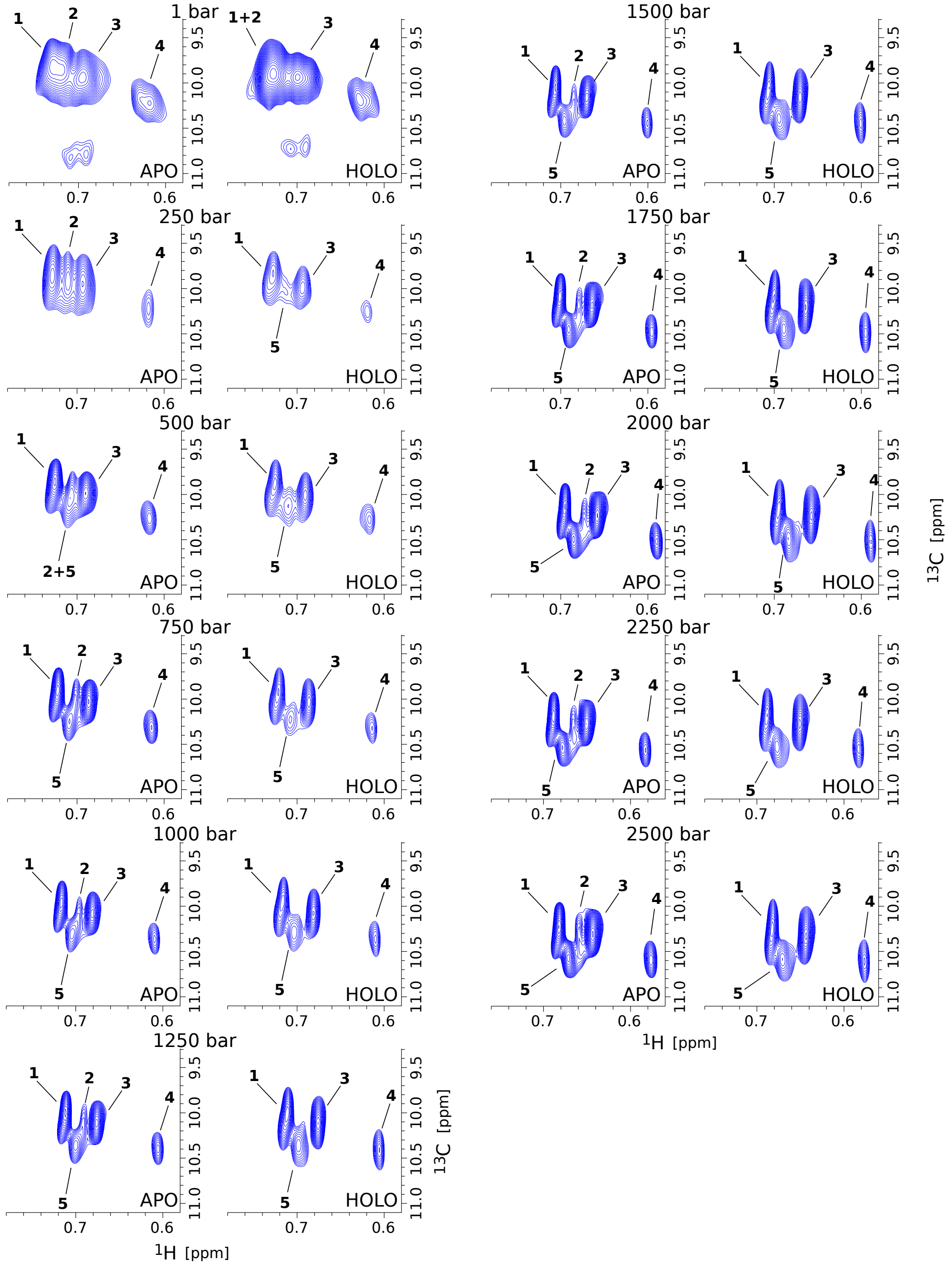

Fig. S12. Comparison between the *apo* and the *holo* states of the evolution of I229 high-populated sub-state NMR signals along the pressure ramp of the receptor in POPC/POPG nanodiscs. Numbers *1* to *5* referred to the populations displayed in Fig. 7 in the main text. The population *2* observed with DPPC nanodiscs in both the *apo* and *holo* states of the receptor (see Fig. S13), but also with POPC/POPG nanodiscs with BLT2 in the *apo* state, disappears in the presence of the agonist, whatever the pressure.

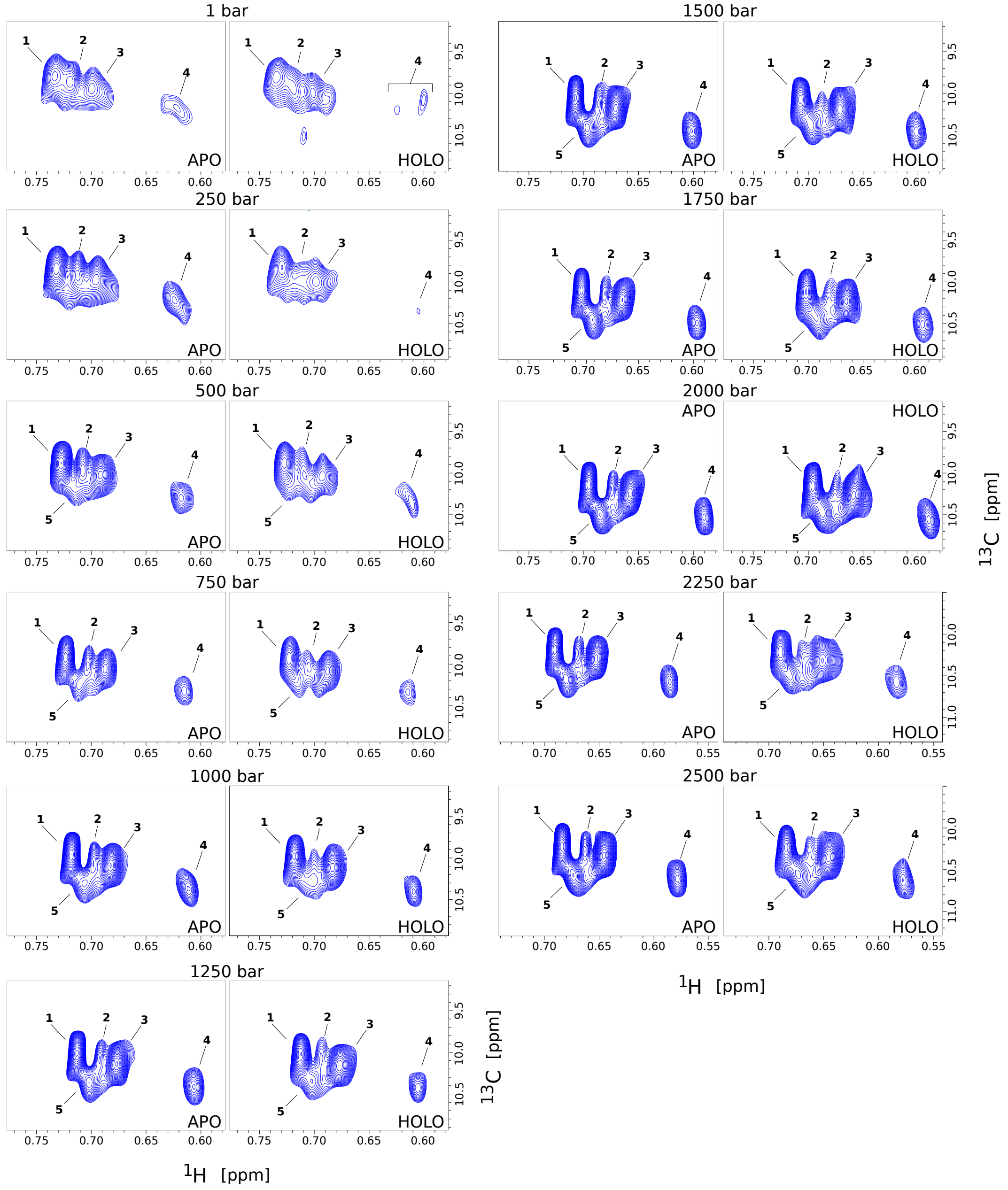

Fig. S13. Same as Fig. S12 but for the receptor in DPPC nanodiscs.

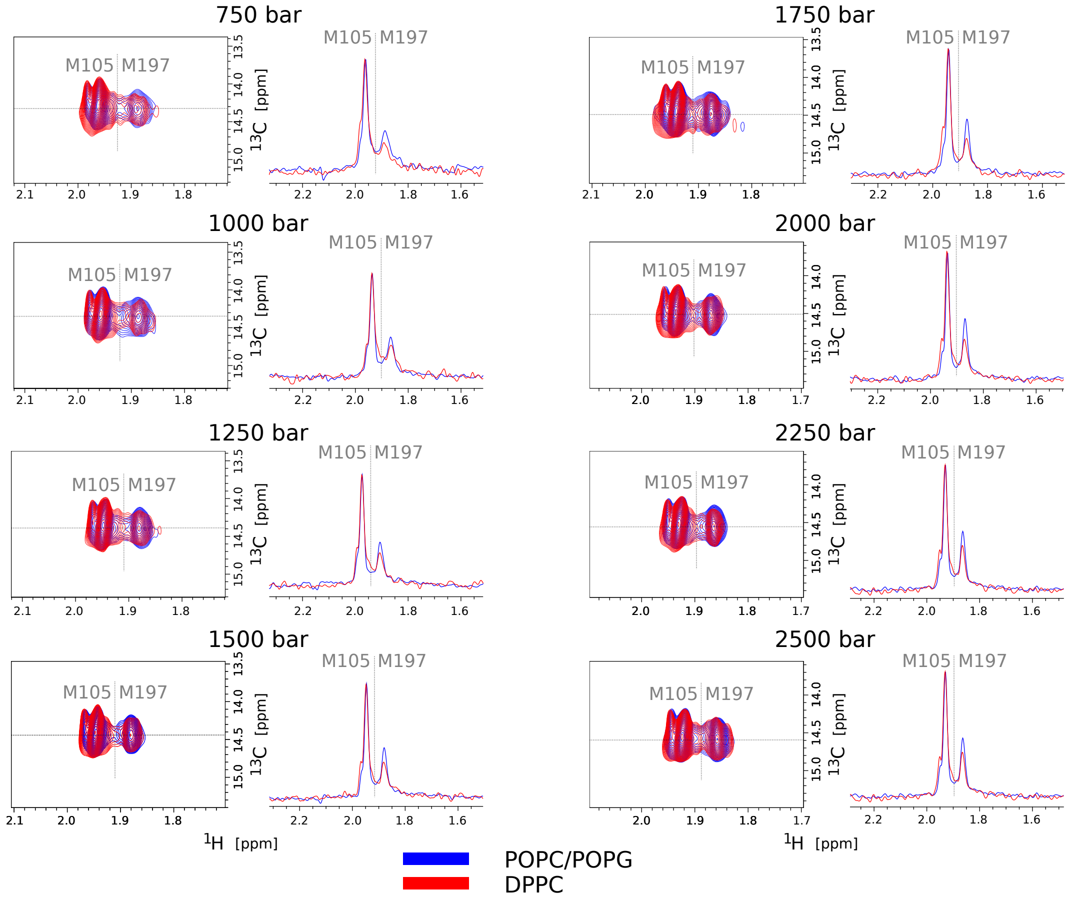

Fig. S14. Comparison of M105 and M197 $^{13}CH_3$–ϵ signals between *apo* BLT2 in POPC/POPG (in *blue*) and DPPC (in *red*) nanodiscs. The 2D superimposed spectra represent ^{1}H,^{13}C SOFAST–HMQC experiments. 1D spectra represented on the right of each 2D experiments correspond to rows extracted along the ^{1}H dimension at the ^{13}C frequency indicated by a horizontal dotted grey line. The vertical dotted line separates the resonances of M105 and M197 in the ^{1}H dimension. The two 1D spectra have been normalized respectively to the highest M105 resonance. Only experiments recorded at pressures greater than 500 bar are compared because at pressures $\leq$500 bar there are too many sub-states and/or the signal-to-noise ratio is too low to properly compare the two sets of data.

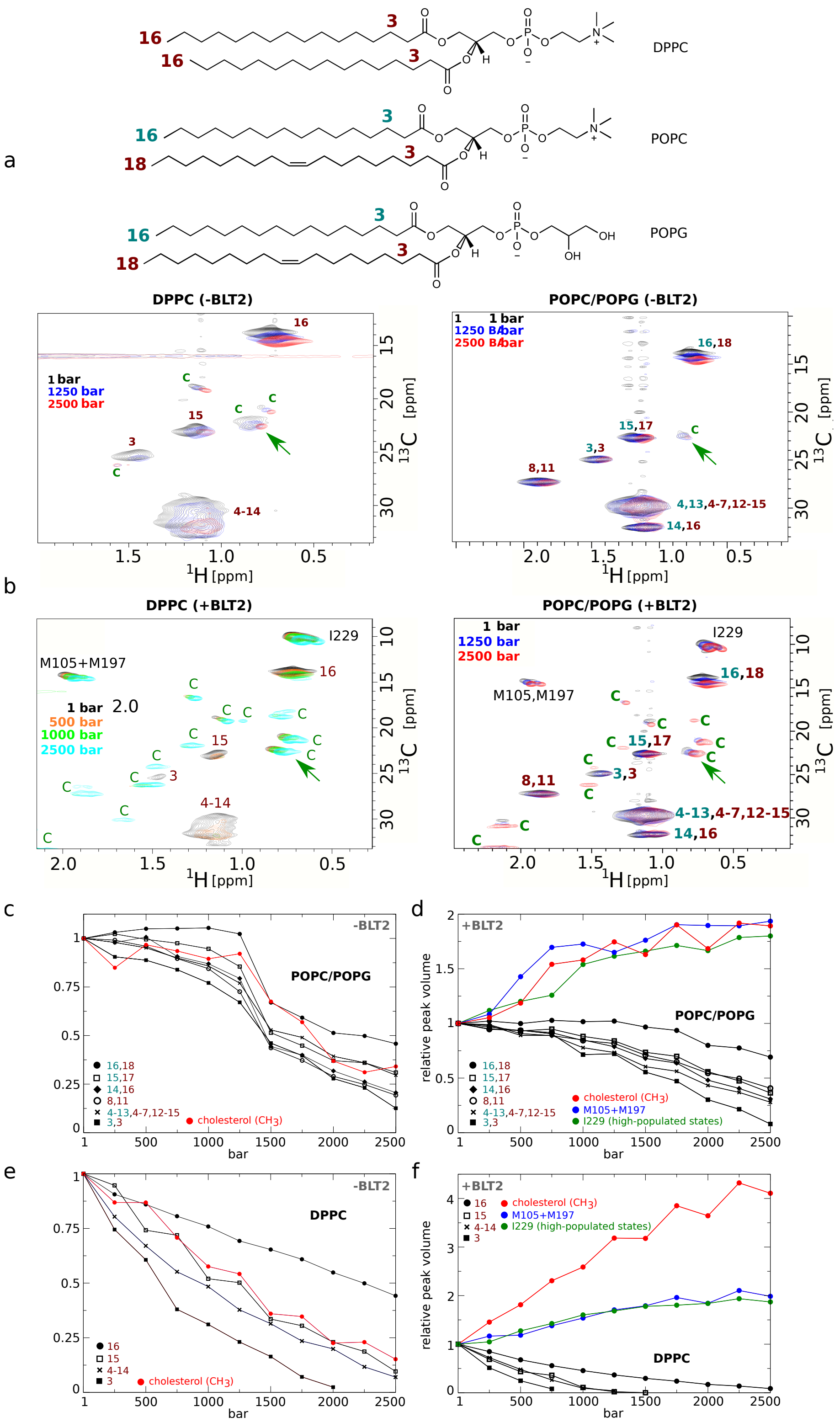
a
DPPC
POPC
POPG
b
DPPC (-BLT2)
POPC/POPG (-BLT2)
DPPC (+BLT2)
POPC/POPG (+BLT2)
1 bar
1250 bar
2500 bar
500 bar
1000 bar
M105+M197
M105,M197
I229
16,18
15,17
3,3
8,11
4,13,4-7,12-15
14,16
4-14
^{1}H [ppm]
^{13}C [ppm]
c
d
e
f
-BLT2
+BLT2
POPC/POPG
DPPC
relative peak volume
bar
cholesterol (CH3)
M105+M197
I229 (high-populated states)

Fig. S15. Barotropic evolution of cholesterol NMR signals in the absence or presence of the receptor. (**a**) Lipid primary structures. The colored numbers are reproduced in the spectra in **b** and in the labels of graphs displayed in **c** to **f**. (**b**) Barotropic evolution at 25°C of the NMR signals of the cholesterol (indicated by the letter C) and $^{13}CH_2$ and $^{13}CH_3$ moieties of DPPC (*Left*) and POPC/POPG (*Right*) acyl chains in receptor-free nanodiscs (*Top*) or in the presence of BLT2 (*Bottom*) observed in 2D ^{1}H,^{13}C–SOFAST–HMQC (950 MHz ^{1}H Larmor frequency) spectra. (**c** to **f**) Comparison of the evolution of one $^{13}CH_3$ cholesterol NMR signal (green arrow in **b**) in lipid discs with POPC/POPG (*Top*: **c** and **d**) and DPPC (*Bottom*: **e** and **f**) acyl chain NMR signals in the absence (*left*: **c** and **e**) or in the presence (*right*: **d** and **f**) of BLT2 receptor along the pressure ramp at 25°C from 2D ^{1}H,^{13}C–SOFAST–HMQC (950 MHz ^{1}H Larmor frequency) NMR spectra. In **c** to **f**, the evolution of the NMR signal of one methyl group of the cholesterol is indicated in *red* (at 0.82/22.4 $^{1}H/^{13}C$ ppm at 1 bar in receptor-free nanodiscs). In **d** and **f**, in *blue* and *green* are also represented the evolution of the global volumes of ϵ-$^{13}CH_3$-[M105$^{3.35}$ + M197$^{5.54}$] and δ_1-$^{13}CH_3$-I229$^{6.40}$ (only I229 highly populated states, see Fig. 7A in the main text), respectively. The volume of reference for each signal has been taken at atmospheric pressure. Source data are provided as a Source Data file.

⟵

3. Additional References

1. S. Balayssac, M.-A. Delsuc, V. Gilard, Y. Prigent, M. Malet-Martino, Two-dimensional DOSY experiment with Excitation Sculpting water suppression for the analysis of natural and biological media. *J. Magn. Res.* **196**, 78–83 (2009).

2. E. O. Stejskal, J. E. Tanner, Spin Diffusion Measurements: Spin Echoes in the Presence of a Time-Dependent Field Gradient. *J. Chem. Phys.* **42**, 288–292 (1965).

3. I. H. Bell, J. Wronski, S. Quoilin, V. Lemort, Pure and Pseudo-pure Fluid Thermophysical Property Evaluation and the Open-Source Thermophysical Property Library CoolProp. *Ind. Eng. Chem. Res.* **53**, 2498–2508 (2014).

4. R. Winter, Pressure Effects on Artificial and Cellular Membranes. *Subcell. Biochem.* **72**, 345–370 (2015).

5. C. Doyen, E. Larquet, P. D. Coureux, O. Frances, F. Herman, S. Sablé, J. P. Burnouf, C. Sizun, E. Lescop, Nuclear Magnetic Resonance Spectroscopy: A Multifaceted Toolbox to Probe Structure, Dynamics, Interactions, and Real-Time In Situ Release Kinetics in Peptide-Liposome Formulations. *Mol. Pharm.* **18**, 2521–2539 (2021).

6. A. Watts, NMR of Lipids. In: G. C. K. Roberts (eds) Encyclopedia of Biophysics. Springer, Berlin, Heidelberg (2013).

7. E. Lescop, T. Kern, B. Brutscher, Guidelines for the use of band-selective radiofrequency pulses in hetero-nuclear NMR: example of longitudinal-relaxation-enhanced BEST-type 1H-15N correlation experiments. *J. Magn. Reson.* **203**, 190–198 (2010).

8. P. Schanda, B. Brutscher, Very fast two-dimensional NMR spectroscopy for real-time investigation of dynamic events in proteins on the time scale of seconds. *J. Am. Chem. Soc.* **127**, 8014–8015 (2005).

9. F. Hagn, M. Etzkorn, T. Raschle, G. Wagner, Optimized phospholipid bilayer nanodiscs facilitate high-resolution structure determination of membrane proteins. *J. Am. Chem. Soc.* **135**, 1919–1925 (2013).

10. A. J. Kooistra et al., GPCRdb in 2021: integrating GPCR sequence, structure and function. *Nucleic Acids Res.* **49**, D335–D343 (2021).
