# Supplementary material for: Exploration of the dynamic interplay between lipids and membrane proteins by hydrostatic pressure
Source: Nat Commun. 2022 Apr 1;13:1780. doi: 10.1038/s41467-022-29410-5 (PMC8975810; doi:10.1038/s41467-022-29410-5)
Supplement: Supplementary file 3 — Reporting Summary [file 41467_2022_29410_MOESM3_ESM.pdf]

nature research

Corresponding author(s): Ewen Lescop & Laurent J. Catoire

Last updated by author(s): Feb 16, 2022

Reporting Summary

Nature Research wishes to improve the reproducibility of the work that we publish. This form provides structure for consistency and transparency in reporting. For further information on Nature Research policies, see our Editorial Policies and the Editorial Policy Checklist.

Statistics

For all statistical analyses, confirm that the following items are present in the figure legend, table legend, main text, or Methods section.

n/a	Confirmed	
☐	☒	The exact sample size (*n*) for each experimental group/condition, given as a discrete number and unit of measurement
☐	☒	A statement on whether measurements were taken from distinct samples or whether the same sample was measured repeatedly
☐	☒	The statistical test(s) used AND whether they are one- or two-sided<br>*Only common tests should be described solely by name; describe more complex techniques in the Methods section.*
☒	☐	A description of all covariates tested
☒	☐	A description of any assumptions or corrections, such as tests of normality and adjustment for multiple comparisons
☐	☒	A full description of the statistical parameters including central tendency (e.g. means) or other basic estimates (e.g. regression coefficient) AND variation (e.g. standard deviation) or associated estimates of uncertainty (e.g. confidence intervals)
☐	☒	For null hypothesis testing, the test statistic (e.g. *F*, *t*, *r*) with confidence intervals, effect sizes, degrees of freedom and *P* value noted<br>*Give P values as exact values whenever suitable.*
☒	☐	For Bayesian analysis, information on the choice of priors and Markov chain Monte Carlo settings
☒	☐	For hierarchical and complex designs, identification of the appropriate level for tests and full reporting of outcomes
☒	☐	Estimates of effect sizes (e.g. Cohen's *d*, Pearson's *r*), indicating how they were calculated

Our web collection on statistics for biologists contains articles on many of the points above.

Software and code

Policy information about availability of computer code

Data collection	TopSpin 3.6 and 4 (Bruker BioSpin); Cary Eclipse software (Varian)
Data analysis	TopSpin 3.6 and 4 (Bruker BioSpin); CCPNMR 2.5

For manuscripts utilizing custom algorithms or software that are central to the research but not yet described in published literature, software must be made available to editors and reviewers. We strongly encourage code deposition in a community repository (e.g. GitHub). See the Nature Research guidelines for submitting code & software for further information.

Data

Policy information about availability of data

All manuscripts must include a data availability statement. This statement should provide the following information, where applicable:

- Accession codes, unique identifiers, or web links for publicly available datasets
- A list of figures that have associated raw data
- A description of any restrictions on data availability

Relevant data supporting the finding of this study are contained within the paper, Supplementary Information, and Source data file.

Field-specific reporting

April 2020

Life sciences study design

All studies must disclose on these points even when the disclosure is negative.

Sample size	No sample size calculations were performed. Experiments were repeated 3 times to allow calculation of the mean and standard error of the mean. Sample size was chosen to obtain statistical significant values.
Data exclusions	No data was excluded from analysis.
Replication	NMR experiments and GTP binding assays were repeated 2 to 3 times. We confirmed that the replication of the measures were successful. All attempts at replication were successful.
Randomization	No randomization was attempted as it was not necessary for these experiments (no animal or clinical case/control study).
Blinding	No blinding was not performed as it was not necessary. In most cases, a given series of data were generated and analyzed by the same individual investigators.

Reporting for specific materials, systems and methods

We require information from authors about some types of materials, experimental systems and methods used in many studies. Here, indicate whether each material, system or method listed is relevant to your study. If you are not sure if a list item applies to your research, read the appropriate section before selecting a response.

Materials & experimental systems

n/a	Involved in the study	
☒	☐	Antibodies
☒	☐	Eukaryotic cell lines
☒	☐	Palaeontology and archaeology
☒	☐	Animals and other organisms
☒	☐	Human research participants
☒	☐	Clinical data
☒	☐	Dual use research of concern

Methods

n/a	Involved in the study	
☒	☐	ChIP-seq
☒	☐	Flow cytometry
☒	☐	MRI-based neuroimaging
